# The Conceptual and Methodological Mayhem of “Screen Time”

**DOI:** 10.3390/ijerph17103661

**Published:** 2020-05-22

**Authors:** Linda K. Kaye, Amy Orben, David A. Ellis, Simon C. Hunter, Stephen Houghton

**Affiliations:** 1 Department of Psychology, Edge Hill University, Ormskirk L39 4QP, UK; 2 Emmanuel College, University of Cambridge, Cambridge CB2 3AP, UK; aco35@cam.ac.uk; 3 MRC Cognition and Brain Sciences Unit, University of Cambridge, Cambridge CB2 7EF, UK; 4 School of Management, University of Bath, Bath BA2 7AY, UK; dae30@bath.ac.uk; 5 Department of Psychology, Glasgow Caledonian University, Glasgow G4 0BA, UK; simon.hunter@gcu.ac.uk; 6 Graduate School of Education, University of Western Australia, 6009 Perth, Australia; stephen.houghton@uwa.edu.au

**Keywords:** screen time, screen use, well-being, social media, self-reports, methods, affordances

## Abstract

Debates concerning the impacts of screen time are widespread. Existing research presents mixed findings, and lacks longitudinal evidence for any causal or long-term effects. We present a critical account of the current shortcomings of the screen time literature. These include poor conceptualisation, the use of non-standardised measures that are predominantly self-report, and issues with measuring screen time over time and context. Based on these issues, we make a series of recommendations as a basis for furthering academic and public debate. These include drawing on a user-focused approach in order to seek the various affordances gained from “screen use”. Within this, we can better understand the way in which these vary across time and context, and make distinction between objective measures of “screen time” compared to those more subjective experiences of uses or affordances, and the differential impacts these may bring.

## 1. Introduction

Debate about the psychological impacts of screen time on young people remains a regular feature of societal conversation. Specifically, concerns that screen time negatively impacts psychosocial functioning, health and behaviours remain widespread, and are routinely voiced in academic, policy and media circles. Whilst some evidence routinely shows negative correlations between screen time and well-being [1,2], recent reviews and meta-analyses repeatedly conclude that the evidence base is difficult to interpret and presents mixed findings [3,4]. In recent years, a growing consensus has emerged suggesting that the associations observed between screen time and psychological or health outcomes are very small in size and that there lacks clear longitudinal evidence that they represent a pure causal effect driven by screen media [5,6,7]. 

Strong evidence concerning the positive or negative impacts of screen time is, therefore, surprisingly sparse. At the same time, other factors tend to explain far more variability when researchers adopt more theoretically-aligned approaches that explain the link between screen time and psychosocial well-being [3,7]. For example, social support garnered through screen-based activities [8], types of social interactions [9], pre-existing levels of social anxiety [10], and fear of missing out [11] might be more influential in predicting well-being outcomes than screen time directly. Further, when quantifying negative outcomes, especially for young people, many other factors continue to remain far more influential. For example, adversity in childhood consistently predicts poor psychosocial functioning and long-term health outcomes [12]. Therefore, screen time sits within a complex mesh of individual and social factors—many of which are already known to have substantial and clear ramifications on everyday functioning, with non-uniform outcomes.

While debate, therefore, continues regarding both the size and importance of screen time associations with health and well-being, basic methodological and philosophical issues surrounding screen time are routinely overlooked. These issues include poor conceptualisation, and the routine use of non-standardised measures that are predominantly self-report. In addition, many research designs rely on cross-sectional data which cannot begin to unpick the causal nature of observed associations between screen time and well-being. This paper considers each of these issues in turn, examines the evidence, provides additional insights, and proposes recommendations to improve the quality of future research and discussion in this area.

## 2. Issue 1—Conceptual Chaos—What Is Screen Time? 

Definitions of screen time vary, which poses a myriad of issues relating to harmonisation, measurement and comparison. The Oxford English Dictionary [13] defines screen time as “time spent using a device such as a computer, television, or games console”, whereas the World Health Organisation’s [14] (p 5) most recent guidelines focus on the issue of sedentary screen time and define it as “Time spent passively watching screen-based entertainment (TV, computer, mobile devices). This does not include active screen-based games where physical activity or movement is required.” The WHO, therefore, does not include screen activities like exergaming, which has been credited with upending the stereotype of gaming as a sedentary activity [15]. Indeed, it has been claimed that current paediatric recommendations specifically surrounding (sedentary-based) screen use may be unachievable based on the fact that screen-based media is relatively central to people’s everyday lives [16]. This highlights the multitude of purposes that screens have in everyday life, serving to support behaviours such as communication, information seeking and entertainment. 

Despite this, there is a tendency to assume that screen time is a largely unidimensional or homogeneous construct, with little work to unify definitions or ensure that conceptualisations are agreed upon. This has led to many different approaches when conceptualising and measuring screen time. For example, a selection of conceptualisations are included in Table 1. 

This diverse nomenclature could be due to the area of study being new, with a unified vocabulary yet to be established by the academic community. However, such diversity is also a clear indication that a concept is relatively undefined even within expert circles. It is, therefore, unsurprising that there is wide variation in the self-report measures used to capture screen time. Whereas some studies have probed largely universal ‘total screen time’ in respect of a certain time-frame (e.g., in the last week), others ask participants to estimate the amount of screen time on a typical day, and sometimes split this by weekday and weekend day, or school day and non-school day. Other studies take different approaches, either asking supplementary measurements of usage by type of device or activity (e.g., passive use/TV viewing, social media use) or focusing more exclusively on one specific technology use or the use of a specific platform or application. This may include questions about attitudes towards technology use that are then inferred as a proxy for screen time, such as the Facebook Intensity Scale [26]. It is unclear whether taking a sum or average of all these different measures equates to asking a participant to estimate their overarching screen time, and there has been little work examining how these different types of measurement relate to each other. 

One noteworthy study, focusing on Facebook use, has explored variations in how participants respond based on the time-frame reference points of screen time questions, and whether these correspond to actual Facebook use [27]. These included time-frames such as minutes per day, time per day, daily time in the past week, and number of times per day. Ernala et al.’s [27] findings illuminated that there are variations in accuracy of responding based on the time-frame reference point—namely, that participants were most accurate (albeit only at a rate of 34%) when reporting on number of times per day they visited Facebook, but very inaccurate when gauging how many “time”, “minutes” or “hours” they had spent on average per day on Facebook (ibid). As such, even simple screen time measures can be expected to include a high degree of measurement error, which is likely to contribute toward contradictory and inconsistent empirical results. (Additional discussion on measurement is provided in Issue 2.)

Developing theoretically valid and practically useful conceptualisations of screen time is likely to only be achieved if users themselves are included as active research participants. Such research is likely to be qualitative in nature and will ideally include users as fundamental partners (e.g., using participatory research methods). This can also inform our understanding of how specific populations may engage with screens. For example, vulnerable populations may have unique uses for screens, as is suggested from work showing that adolescents with neurodevelopmental disorders have higher rates of social media use [28] and may use screens to support social and communication skills [29]. Such advantages can include the fact that these experiences can be highly motivating, with integrated rewards, close control of learning rate and content and personalisation to the individual’s own preferences [30]. These nuances should be integrated into the academic understanding of the concept to make it truly meaningful. This enhanced conceptual understanding of screen time may conclude that the term is simply not a useful concept for research or enhancing public understanding. Thus, we believe that it is fundamentally important for scholars to meaningfully engage with issues surrounding the conceptualisation and measurement of screen time. 

Recommendations:

1. Employ a wider range of user-focused research methodologies to inform a clear and unambiguous conceptualisation of screen time.
2. Include in conceptualisations a differentiation between screen time as a numerical measurement (e.g., minutes per day) and “screen use” as goal-directed behaviour (e.g., to fulfil different motivations such as social use and educational use).

## 3. Issue 2—Estimates Are Not Good Enough

Studies investigating screen time (or any variation of terms on this) most often use users’ estimated reports, either retrospectively or throughout the day. Participants may be asked questions such as “How many hours of screen time did you use in a typical day last week?”, yet it is far from clear whether participants can accurately estimate the amount of screen time they engage in or whether this estimate is systematically biased by screen use itself. Biases may include underestimation by high users, overestimation by low users, developmental issues relating to the accuracy of time estimation itself [31]. That is, accumulating evidence indicates that timing functions are impaired in a variety of developmental disorders including ADHD [32,33], dyslexia [34], and autism [35,36]. 

Whilst self-report measures are commonplace in psychological research, evidence suggests that these can be particularly ill-fitting for the purposes of gaining accurate data on people’s technology-related use [27,37,38]. Namely, when comparing smartphone users’ estimates of their own smartphone use, and checking behaviours with their actual use (garnered from smartphone data), they tend to underestimate these behaviours [39]. Correspondingly, for social networking sites such as Facebook, compared to actual use garnered from log data, people tend to overreport how much time they spend on Facebook but underreport how many times they visit per day [27]. This appears to become even more problematic when estimating short, habitual screen time behaviours like smartphone checking [39], as highlighted in recent work showing that iPhone users’ estimates of daily social media use are less accurate than their reports of overall daily iPhone use [38]. In this context, developments such as the Apple screen time App allow researchers to gain objective measurements of behaviour surrounding smartphone use [38,40]. However, challenges remain when wishing to record screen time on other platforms such as laptops or TVs or screen time on shared devices (e.g., family consoles or a university PC). This issue is discussed further in Issue 3. 

It is perhaps unsurprising that there has been little movement in the field to develop high-quality screen time measures because this involves a variety of methodological, security and privacy challenges. For example, the Human Screenome project [41] aims to unobtrusively collect screen recordings which show moment-by-moment changes on users’ screens. However, notwithstanding security and privacy concerns, resources are not freely available that would allow other researchers to take advantage of these methods. These developments do not diminish the importance of subjective experiences but measurements should be orientated with the specific research question, which, in this case, typically aligns with a focus on usage alone.

Recommendations:

1. Make use of objective screen data such as data logs, screen time apps which record the use of apps of interest, and server data where possible (e.g., Apple screen time, which records the use of apps based on categories such as “entertainment” and “health and fitness”).
2. Encourage and contribute to transdisciplinary programs of work around privacy, security and methodological challenges relevant to the adoption of objective measurement.
3. Work to develop measures of screen time should seek to develop scales and assessments which are as brief as possible, especially when these are designed to assess children and young people’s screen time.

## 4. Issue 3—Screen Use Varies across Contexts

While recording screen time on individual devices such as a smartphone, or a specific smartphone app is now possible, even these approaches will struggle to address how much time is spent on different forms of screen-based engagement which take place on multiple screens. For example, social media can be accessed via a smartphone, tablet, TV, games console, and/or PC. It seems unlikely that data can be harvested from all of these concurrently, especially when at least some will be shared with people other than a study participant (e.g., a family TV). In addition, people engage with screens that are not necessarily their own (e.g., a friend’s phone and a school laptop) and this further limits the utility of approaches based on device-level records of actual use. There are of course economic aspects to consider in relation to this issue, and the extent to which different users will have the means and access to varied types of technology in different places. 

The issue of multitasking overlaps with that of screen proliferation. Imagine a researcher wants to record how much screen time a person engages in between 19.00 and 20.00. The participant has a TV on for the whole hour, uses a laptop for 25 minutes to complete some work, and checks their social media whenever they get an alert on their smartphone. How should the person report their screen time? Previous work on media multitasking (changing across devices) and task switching (changing channels or task within the same device) has been explored quite extensively in the literature, but largely in respect of how this impacts on cognitive or attentional capacity [42]. In general, these have tended to define media multitasking as simultaneous media usage via several devices [43,44]. However, these studies have failed to reach a reasonable consensus on how best to measure the proportionality of the use of specific devices or channels during these instances. 

As previously noted, the available literature on screen time tends to use either a score of one’s total “screen time” or a number of such scores by type of device or activity. Whilst the latter of these is helpful to ascertain more clearly how certain types of screen activities may impact differentially on outcomes, what is currently omitted using this approach is how the various combinations of different screen time activities (which may occur across contexts) function in this regard. That is, someone with a high proportion of entertainment screen use (watching TV) relative to other screen-based activities (e.g., social media use) may be differentially impacted compared to someone with a more equally-weighted “screen time profile”. Any latent-based measure may need to be cognizant of other important socio-cultural confounds including economic status. However, no research to date has used a profiling or cluster-based approach to explore how these various screen time proportionalities may map out onto psychological or behavioural outcomes. This would be a helpful advancement within the field, and potentially presents a more holistic approach to the issue than current approaches.

Recommendations:

1. Develop taxonomies to provide a conceptual basis for mapping different types of screen use and associated behaviours. This can help ascertain how proportionality of different types of use may relate differentially to psychological outcomes.

## 5. Issue 4—Screen Use Changes over Time

The majority of research on screen time has obtained cross-sectional data to understand the relationships with psychological and behavioural outcomes. While such cross-sectional research is important in an effort to examine a fast moving target, and where there might not be enough time to collect longitudinal data, it inherently limits the scope of conclusions. A handful of studies that have approached questions around screen time using longitudinal methods observe either no or very small effects for the impact of screen time on experiences such as depressive symptoms [21,45]. This holds when exploring specific forms of screen activities such as video games [46], social media [5,6,47,48], and passive screen use [21]. 

While longitudinal research is invaluable for beginning to establish causal relationships, it brings with it a set of unique concerns. Longitudinal research, by its nature, requires data to be gathered at multiple time-points, which can extend over a period of months or often years. There are three major challenges facing researchers who wish to conduct such studies. First, it is unclear how applicable any given measure of “screen time” will be for the duration of a given piece of research. For example, if screen time is recorded as a platform- or device-specific activity (e.g., Facebook use or iPhone use) then the measurement is dependent upon the success and durability of those platforms/devices. Second, the degree to which different cohorts use comparable platforms or devices in comparable ways is far from clear. For example, the way in which a 12 year old uses the most up-to-date iPhone in 2021 will not necessarily be directly comparable to how a 12 year old uses the most up-to-date iPhone in 2024; the capabilities and affordances of the 2024 model may make it a fundamentally different device to the 2021 model. In this rapidly evolving environment, documenting cohort-specific screen use is of paramount importance. Finally, work with children and young people often involves data collection within schools and during class time. As such, repeated data collection can become disruptive for participants’ education and this can make it more likely that researchers will seek out measurements that are quick but which are less psychometrically substantive to draw strong conclusions.

One solution is to move away from measuring the platform use itself, and instead measure affordances/activities obtained via platforms. For example, video-chatting is a social activity which is mediated by a number of different platforms such as Facebook Messenger, Zoom and FaceTime. An affordance-based approach avoids relying on the commercial success of platforms or devices and instead establishes measurements which should theoretically remain more stable over time. This can support researchers to ensure that responses are comparable. Failure to attend to this fundamental measurement issue is equivalent to asking participants how they interact with novels at the start of a study and then asking them how they interact with newspapers later. 

In addition to comparable longitudinal measurements, better use of high-quality experimental studies is needed to probe causal links between screen time and outcomes of interest. However, studies of this nature would necessitate the use of control groups where subsets of participants are asked to refrain from using screens (or a certain subset of them). Such studies are lacking because ensuring participant compliance is challenging [4,49], and those published fail to show consistent results [50,51,52]. However, a more viable way forward may be to form control groups based on restrictions to certain features of technology (e.g., social media sites or apps) for a given time-frame to compare with those using these features as normal. Despite control groups being considered part of the gold-standard approach within effective scientific design, there is a scant attention to this issue in current screen time literature. To our knowledge, even the obvious question of who operates as a control group in this type of research has not yet been fully appreciated. A true control group in this case would be those who are abstinent from all screens for a predetermined time-frame, yet this would be almost impossible for large sections of society, as screens serve a multitude of everyday functions and are embedded within our schools and workplaces. Within the literature, these questions have simply not been asked, and therefore present additional methodological uncertainty for those working in this area. Although removing screens entirely from people’s lives is not viable as a form of control group, some work has measured the effects of smartphone abstinence (for up to 24 hours) on short-term outcomes such as mood, anxiety and craving [53]. Alternatively, researchers may also start to realise new opportunities by teasing out causal relationships between screen time and psychological impacts with observational data alone [54].

Recommendations:

1. As per recommendation 2, defining screen time instead in reference to screen use of more specified, targeted behaviours (e.g., social screen use) is focused more on the behaviour itself rather than the technological affordances available at certain time-points. If measurements are more centrally developed around the behaviours screens are affording rather than what “functions and features” people are using, this is less volatile as a reference point and less likely to result in comparability issues across time-points.
2. Control groups should feature more prominently in research paradigms, such as including people who are restricted from using certain screen activities for engaging in certain behaviours for a designated time-period.

## 6. Conclusions

There are a number of complex and nuanced issues in work addressing screen time and its outcomes. These include the difficulties conceptualising screen time, limitations in self-report data, and challenges for measuring screen time over time and in different contexts. Some of these issues may be rectified at least in part by the use of more objective-driven approaches rather than estimates of behaviour. This is certainly an important avenue to advance this field, and a key recommendation for improving scientific insight into these issues. However, this is only a partial solution to a broader conceptual debate. As a first step, we reiterate what others have already recommended [55,56]: that the term “screen time” is retired. Instead, alongside our recommendations outlined above, we propose an affordance-based approach as a logical and useful step forward. Here, “screen use” (irrespective of platform or feature) is based on the behaviour(s) screens are facilitating, which may relate to behaviours such as entertainment, social, education/work, and informational. To structure such an approach, the needs of users should be the central concern and users themselves should be co-creators of this, through qualitative work which listens to the voice of these various users. This should be the next step to further academic and public debate around conceptualising and measuring screen use, so that we may be better equipped to explore its psychological and social correlates.

## Figures and Tables

**Table 1:** Different terminology used in screen time work.

Term	Examples of Authors
Sedentary screen-based behaviour	Suchert et al. [17]
Media time	Stockdale et al. [18]
	Twenge and Campbell [19]
Screen time	Ferguson [20]
	Houghton et al. [21]
	Wu et al., [22]
Screen use	Ferguson [20]
	Houghton et al. [21]
	Wu et al. [22]
New media screen time	Twenge et al. [23]
Digital media time	Twenge and Campbell [19]
Technology use	Nesi and Prinstein [24]
Digital engagement	Orben and Przybylski [25]
Digital technology use	Orben and Przybylski [7]
